# Modification and completion of geological structure knowledge graph based on pattern matching

**DOI:** 10.1038/s41598-024-60618-1

**Published:** 2024-04-29

**Authors:** Cai Lu, Xinran Xu, Bingbin Zhang

**Affiliations:** School of Information and Communication Engineering, University of Electronic and Science Technology of China, Chengdu, China

**Keywords:** Geological structure knowledge graph, Patterning mining, Graph mining, Sub-graph matching, Geology, Geomorphology

## Abstract

As a knowledge representation method, knowledge graph is widely used in intelligent question answering systems and recommendation systems. At present, the research on knowledge graph mainly focuses on information query and retrieval based on knowledge graph. In some domain knowledge graphs, specific subgraph structures (patterns) have specific physical meanings. Aiming at this problem, this paper proposes a method and framework of knowledge graph pattern mining based on gat. Firstly, the patterns with specific physical meaning were transformed into subgraph structures containing topological structures and entity attributes. Secondly, the subgraph structure of the pattern is regarded as the query graph, and the knowledge graph is regarded as the data graph, so that the problem is transformed into an approximate subgraph matching problem. Then, the improved relational graph attention network is used to fuse the adaptive edge deletion mechanism to realize the approximate subgraph matching of subgraph structure and attribute, so as to obtain the best matching subgraph. The proposed method is trained in an end-to-end manner. The approximate subgraph matching is realized on the existing data set, and the research work of key pattern mining of complex geological structure knowledge graph is carried out.

## Introduction

With the increase in the amount and diversity of data available for knowledge graphs (KGs)^1^, there is a growing need to analyse them and understand their content. The schema layer is provided by schemas (RDFS), ontologies (OWL) and constraints (SHACL and ShEx). These are logical statements that express absolutely true information about the data and usually need to be presented by a human expert^2^. In this paper, we are interested in knowledge that lies between raw facts and semantics, which are the key patterns of knowledge graphs. These key patterns can serve as indicators of regularities in the data, which in turn can be expressed as patterns or constraints, allowing the user to improve the modeling of KG^3,4^. Or one can help optimise query evaluation by highlighting different prototypes of entities. Moreover, since patterns are extracted from raw facts, they can highlight errors present in the data or modelling when they do not conform to the expected pattern^5^.

Graph structure is ubiquitous in the real world, and many studies have found and exploited repeated subgraph patterns in the input graph. These subgraph patterns are also known as network motifs on isomorphic graphs^6^ or a meta-structure on a heterogeneous graph^7,8^. Mining key pattern queries is an important task in the field of knowledge graphs. These key patterns can help us find the potential regularities and associations in the knowledge graph, so as to better understand the information in the knowledge graph. In recent years, approximate subgraph matching algorithms have been widely used in key pattern mining tasks in knowledge graphs. This algorithm can find other concepts or relationships related to a concept by finding similar subgraphs in the knowledge graph. We model the problem of graph pattern mining in knowledge graphs as an approximate subgraph matching problem of knowledge graphs^9^ for mining frequent patterns in knowledge graphs

Graph matching is the process of determining the compatibility of node characteristics and graph structure, as well as finding equivalent nodes between graphs while respecting the compatibility of node characteristics and graph structure^10^. It is essential in various real-world applications, including identifying equivalent entities between knowledge graphs (KGs)^11–14^. To improve the abstraction of node features for matching, training GNNs in supervised or semi-supervised models has become the standard approach^15–17^. However, there are three main challenges when performing approximate subgraph matching in the knowledge graph. The query graph and the target graph of approximate subgraph matching differ significantly in size. This is because the query graph is typically much smaller than the target graph, owing to the large candidate space^18^. Additionally, training GNNS is computationally expensive due to the exponential increase in the number of neighbors with depth. Methods such as Ullman^5^, VF2^6^, Ceci^7^, FilM^8^, and VELSET^19^ all have exponential time complexity in the worst case. In real-world graphs, the number of nodes is large, making exact matching time-consuming. Additionally, real graphs are often noisy, which may result in the data graph not containing the exact matching subgraph, and the calculation taking a long time without returning any results. The balance between node, edge, and structure features is crucial for improving the accuracy and robustness of approximate subgraph matching.

To solve the subgraph matching problem efficiently in a noisy background, a fast and imprecise method is required. Graph representation learning methods such as GNN^20,21^, GCN^22^, graphSAGE^23^, and GAT^24^ can be used. These methods map high-dimensional structural data to a low-dimensional embedding space and represent graphs, edges, and nodes with low-dimensional embeddings^14^. While approximate graph matching focuses on determining the compatibility of node and edge features as well as graph structure, graph matching aims to identify equivalent nodes between graphs while also considering the compatibility of nodes, edge features, and graph structure. Knowledge graphs currently in use combine both structure and attributes to identify equivalent entities between them. The use of a knowledge graph embedding model and a relation-aware graph neural network allows for the learning of heterogeneous graphs, improving the abstraction of node features for matching. The training of GNNS in supervised or semi-supervised models has become the standard. Multi-layer GCNS are used to embed information about entities and attributes into low-dimensional vectors, with the aim of achieving equivalent entities as much as possible^25,26^.

There are two approaches to key pattern queries on knowledge graphs. Pattern mining finds unusual structures on a global scale, such as quasi-cliques, bipartite cores, or dense blocks in the adjacency matrix of a graph. Feature learning mainly uses Graph Neural Networks (GNNs) to aggregate local neighborhood information into node representations. The existing learning-based approximate subgraph matching method ignores the edge label. The edge matching mechanism is added to achieve approximate matching of the knowledge graph. During the query and matching process, the existing social network and knowledge graph may encounter edge feature mismatch. To enhance the precision of approximate subgraph matching, there are techniques to improve its robustness by taking into account both semantic and structural similarity^13^. The structure's similarity is ensured through the use of edge pruning techniques. Our contributions are as follows:

Our proposal suggests a process for Key Pattern Mining in a geological structure knowledge graph to extract particular subgraph patterns in the knowledge graph. This will enhance the efficiency of knowledge interaction.

We propose an innovative strategy for approximate subgraph matching to mine key patterns. This strategy takes into account the structural features of nodes, edges, and subgraphs. It uses an adaptive edge deletion mechanism and a GAT feature fusion mechanism to achieve approximate subgraph matching of knowledge graphs and mine frequent patterns.

We verify the effectiveness of our method in four existing datasets of approximate subgraph matching, and mine the key patterns of domain knowledge graphs in practical applications to guide vertical applications.

## Materials and methods

### Pattern mining of KG

Pattern mining is an essential task in data mining, with the goal of discovering valuable patterns, regularities, or associations from large-scale datasets^27^. In the context of knowledge graphs, key pattern mining plays a vital role. Knowledge graphs represent and organize knowledge in the form of graphs, where nodes represent entities or concepts, and edges depict relationships between entities. By analysing key patterns within knowledge graphs, we can uncover significant and influential patterns, which further deepen our understanding of the relationships and structures can be unconvered. The combination of key patterns with approximate subgraph matching allows for the search for subgraphs in the knowledge graph that resemble the key patterns. This mining approach enables the identification of essential subgraphs in the knowledge graph that display similar associations or structures, enabling the discovery of valuable knowledge and information^28^.

In the field of geology, pattern mining techniques can be used to discover significant geological layer patterns or rock type patterns from geological knowledge graphs. By mining these patterns, distinct characteristics of geological layers in different regions or geological periods can be identified, providing valuable insights into the evolution of geological layers and the distribution of rock types. Furthermore, pattern mining techniques can be employed to search for significant subgraphs in the knowledge graph with similar structures or relational patterns.This can reveal regularities in geological structures or distribution patterns of underground resources. The use of a complex geological structure knowledge graph in 3D geological structure modeling has been well established,and it can provide precise constraints for oil and gas exploration^29^. However, it is important to determine whether the knowledge graph in the field contains geological structure patterns that align with expert cognition. To achieve this, it is necessary to use approximate subgraph matching to identify the key patterns within the knowledge graph and make any necessary modifications to correct any inaccuracies. Incorporating a knowledge graph of complex geological structure the accuracy and reliability of geological structure modeling. Representing geological information in a structured graph format, enable the capture of relationships and dependencies between different geological elements. This helps to constrain the modelling process and ensures that the resulting geological structures are consistent with the available knowledge.

### Graph neural network for subgraph matching

Sub-GMN^30^ uses a learning-based graph matching technique that constrains the node-level embeddings of corresponding nodes to approximate each other. However, this assumption is not always valid as the node in the data graph may have additional edges, and the corresponding edge node could contain label information, making the corresponding node a distinct entity. The objective of Sub-GMN to integrate GCN and NTN for congruent node-level embeddings may compromise performance, as it forces different entities to converge within the representational space. Several alternative approaches, such as AEDNet^31^, RDGCN^32^, and NeuralMatch^33^, have been developed. Notably, the Relation-aware Dual Graph Convolutional Network (RDGCN) adeptly captures and combines relational information. RDGCN enhances edge representations by employing a graph attention mechanism through an interaction between the original graph and its dual relational graph. AEDNet, on the other hand, focuses on eliminating superfluous edges to ensure matching that is congruous with structural attributes. These methodologies offer more nuanced and efficacious approaches for graph matching, taking into account the intrinsic complexity and heterogeneity of graph data.

## Problem definition

### Subgraph matching and matching matrix

#### Definition 1

(*Approximate subgraph matching*): A graph is represented as a tuple $(V,E,v\_f,e\_f)$, Where V represent the data graph node set, and the E represent the query graph. Given a labeled data graph $G = (V_{G} ,E_{G} ,v\_f_{G} ,e\_f_{G} )$ and a labeled query graph $Q = (V_{Q} ,E_{Q} ,v\_f_{Q} ,e\_f_{Q} )$. where the represent the $e\_f$ and $v\_f$ represent the node and edge attribute in graph.

#### Definition 2

The Matching Matrix delineates the node-to-node correspondence between the query graph and the target graph. It is defined as follows:

1 $$M_{ij} (G,Q) = \left\{ \begin{gathered} \begin{array}{*{20}c} {\begin{array}{*{20}c} 1 & {m_{n} = j} \\ \end{array} } \\ \end{array} \hfill \\ \begin{array}{*{20}c} 0 & {\begin{array}{*{20}c} {m_{n} \ne j} & {n = 1,2,...k} \\ \end{array} } \\ \end{array} \hfill \\ \end{gathered} \right.,\quad M = [M_{ij} ]_{{|G| \times |G^{\prime } |}}$$

where i and j represent the ith row and jth column of matrix $M_{ij} (G,Q)$, which are associated with i nodes of query graph and j nodes of target graph respectively. $|G|$ and $|G^{\prime } |$ represent the query counts the number of nodes in a graph and data, matching matrix M contains all the matching relation between node to node.

### Graph attention network

Graph Attention Networks (GATs) are neural networks that are specifically designed for processing graph-structured data. The key feature of GATs is their attention mechanism, which helps in assigning different importances to different nodes in a neighborhood. This allows GATs to focus on the most relevant parts of the input graph for a given task:

2 $$\alpha_{v,u} = \frac{{\exp (Leaky\;{\text{Re}} \;LU(\overrightarrow {a}^{T} [Wh_{v} ||Wh_{u} ]))}}{{\sum\limits_{w \in N(v)} {\exp (Leaky\;{\text{Re}} \;LU(\overrightarrow {a}^{T} [Wh_{v} ||Wh_{u} ]))} }}$$

where W is learning weight matrix, instrumental in linearly transforming the feature representations | | said concatenation operation, LeakyReLU represents a variant of the ReLU activation function, characterized by allowing a small, non-zero gradient when the input is negative,$\overrightarrow {a}^{T}$ is a learnable attention vector.

## Model: relational perceptual graph attention network

### Adaptive edge pruning mechanism

**Figure 1:**
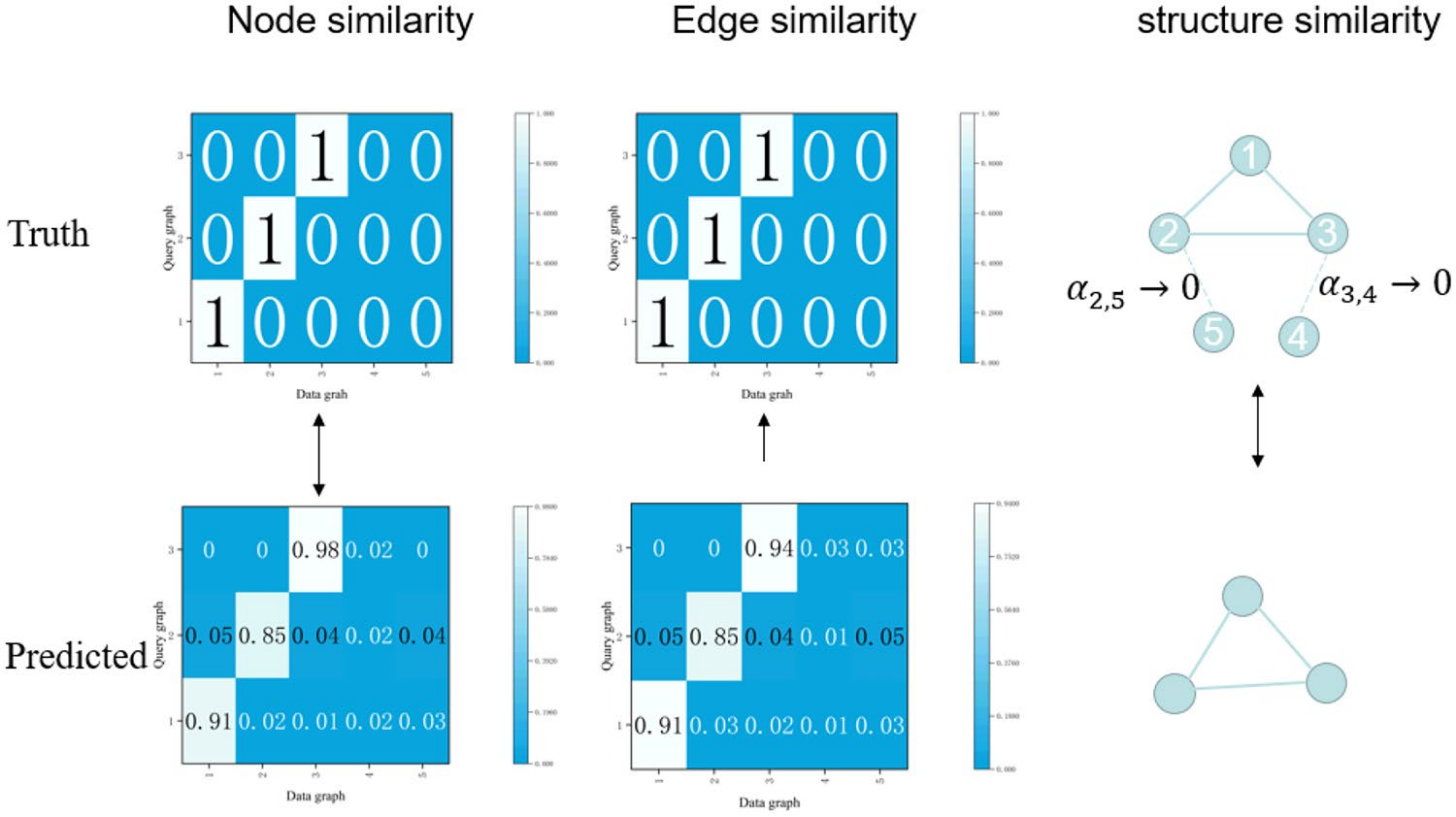
Approximate subgraph matching. For the structural information of nodes and edges that do not match, we want their weights to approach 0, so that the matching matrices of nodes and edges are closer.

Motivated by the challenges outlined above, we introduce a novel Relational Graph Attention Network tailored to execute approximate subgraph matching. As is showed in Fig. 1, this innovative architecture synthesizes an edge pruning methodology with a relational graph attention mechanism. The integration ensures that matching nodes are evaluated based on both their feature representations and adjacency structures, placing emphasis on the congruence of node labels, edge labels, and structural information.

3 $$h_{v}^{k + 1} = MLP^{k} ((1 + \varepsilon^{k} )\alpha_{v,u} h_{v}^{k} + \sum\limits_{u \in N(v)} {h_{u}^{k} } )$$

In order to prevent overfitting of the model, regularization operation can be performed on the new feature vector to obtain the final feature vector of the nodes:

4 $$h_{v}^{k + 1} = LayerNorm(h_{v}^{k + 1} + h_{v}^{k} )$$

5 $$\overrightarrow {a}^{T} = Pooling(H_{Q}^{t} )$$

where $h_{v}^{k}$ represe*nt the node-level embedding at layer t*

6 $$\alpha_{v,u}^{G,(t)} = \frac{{\exp (Leaky{\text{Re}} LU(\overrightarrow {a}^{T} [Wh_{v}^{G} ||Wh_{u}^{G} ]))}}{{\sum\limits_{w \in N(v)} {\exp (Leaky{\text{Re}} LU(\overrightarrow {a}^{T} [Wh_{v}^{G} ||Wh_{u}^{G} ]))} }}$$

7 $$\alpha_{v,u}^{Q,(t)} = \frac{{\exp (Leaky{\text{Re}} LU(\overrightarrow {a}^{T} [Wh_{v}^{Q} ||Wh_{u}^{Q} ]))}}{{\sum\limits_{w \in N(v)} {\exp (Leaky{\text{Re}} LU(\overrightarrow {a}^{T} [Wh_{v}^{Q} ||Wh_{u}^{Q} ]))} }}$$

where $\alpha_{v,u}^{G,(t)}$ and $\alpha_{v,u}^{Q,(t)}$ are normalized attention coefficients for the data graph G and the query graph Q respectively.

In order to get the same neighborhood structure as the query graph and the target graph, we want $$\sum {a_{de}^{G,(t)} = 0}$$$$\left( {A_{u}^{Q,t} = A_{d(v)}^{G,(t)} } \right)$$, We design an adaptive edge deletion loss function:

8 $$L_{D} = \frac{1}{Q}\sum {||a_{ud} - a_{de} - 1||}_{2}$$

where $L_{D}$ ensure that the structure is the same as the original structure after removing the extra.

### Matching mechanism

Different from the previous graph matching methods, we realize the approximate subgraph matching research by adding the information fusion mechanism of edge labels. The fusion mechanism used by the two is the same, and the node features and edge features are matched through the relationship graph attention network

9 $$M_{ij} = \frac{{\exp (s_{h} (h_{i}^{Q,(t)} ,h_{j}^{G,(t)} )*\mu^{ - 1} )}}{{\sum\limits_{j} {\exp (s_{h} (h_{i}^{Q,(t)} ,h_{j}^{G,(t)} )*\mu^{ - 1} } }}$$

where the $$M_{ij} = \{ M_{ij}^{E} ,M_{ij}^{N} \} ,i = 1,...,\begin{array}{*{20}c} N & {j = 1,...,E} \\ \end{array}$$, Represents a normalized match matrix between entities, N and E represent the number of nodes in the query graph and data graph, respectively,$s_{h}$ represents the similarity matrix between entities

10 $$L_{v} = \frac{1}{{N_{Q\_v} }}\sum {||OP - M_{ij}^{N} ||}$$

To ensure a match between edge features, OP represents the matrix of the original match, $M_{ij}^{N}$ Represents the final obtained matrix for evaluating edge features, we hope the $L_{v}$ be less small:

11 $$L_{e} = \frac{1}{{N_{Q\_e} }}\sum {||EP - M_{ij}^{E} ||}$$

where the EP represents the feature matching matrix of the original node, $M_{ij}^{E}$ represents the final obtained matrix for evaluating edge features, we hope the $L_{e}$ be less small.

### Loss function design

Figure 3 utilises prior knowledge as the query graph in deep learning to improve the detail and accuracy of key pattern mining in knowledge graphs and enhance the effect of key pattern queries. This allows for the identification of pattern information in existing data graphs. The loss function design consists of three parts: node features, edge features, and structural attributes.

12 $$L^{t} = \alpha L_{e} + (1 - \alpha )L_{D}$$

13 $$L_{total} = \beta L_{v} + (1 - \beta )L^{t}$$

where $L^{t}$ is the loss at the t-th layer, $\alpha \in [0,1]$ and $\beta \in [0,1][0,1]$ are the hyperparameter that regulates the tradeoff between two components, and the $L_{total}$ is used to balance the two mechanisms.

### Application on real data

**Figure 2:**
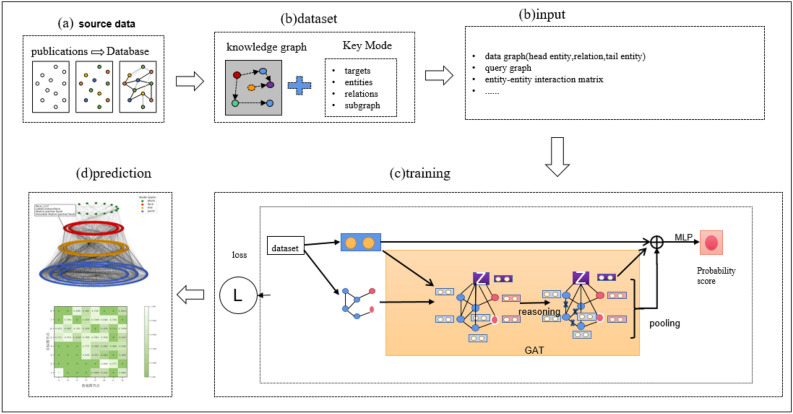
The process of mining key patterns from a knowledge graph involves inputting the query subgraph constructed by expert knowledge, along with the query graph and target graph. The trained model is then used to perform approximate subgraph matching of the knowledge graph, which improves the efficiency of expert interaction in the field of geological structure and promotes research into complex geological structure oil and gas pools.

**Figure 3:**
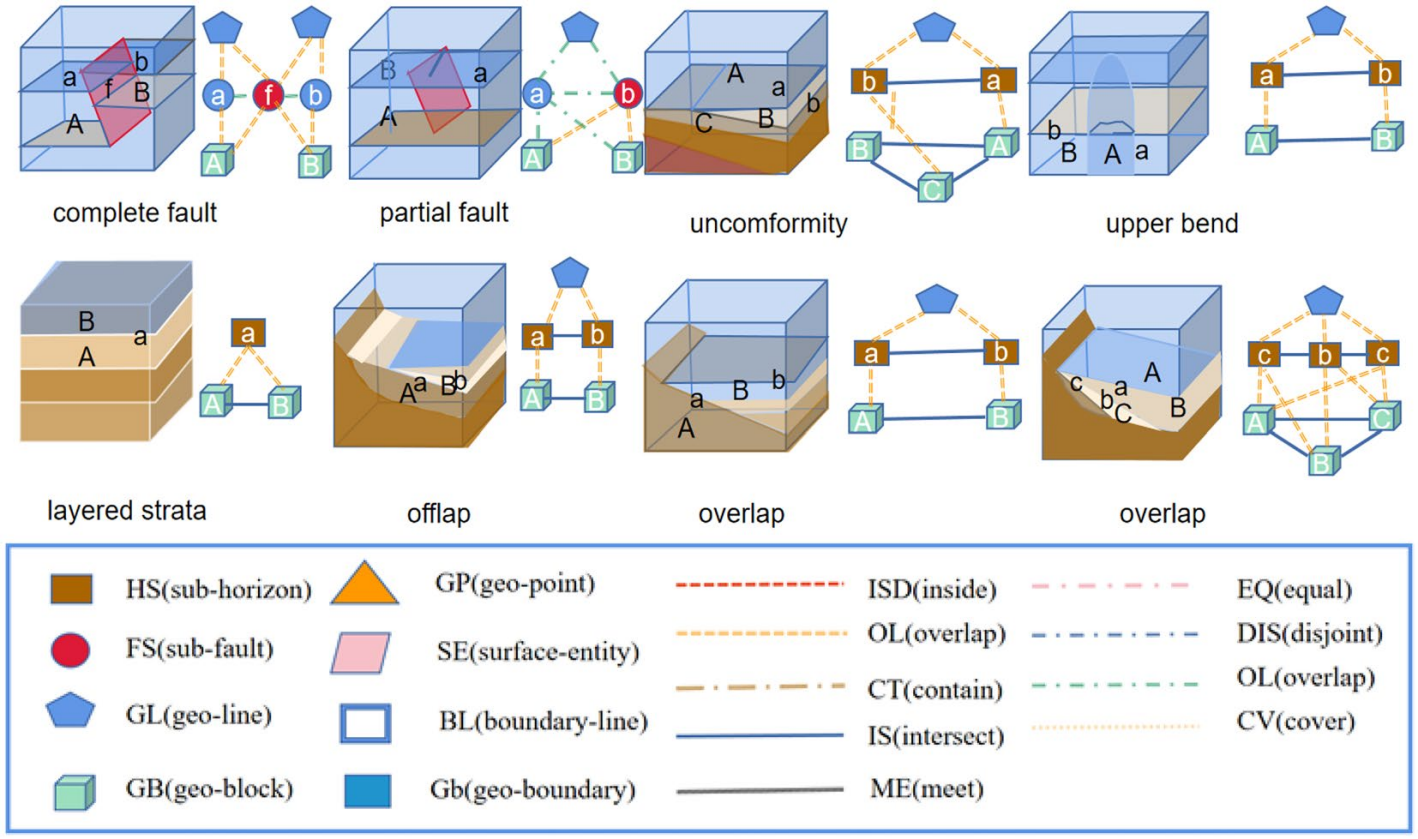
Knowledge graph query graph pattern.

**Figure 4:**
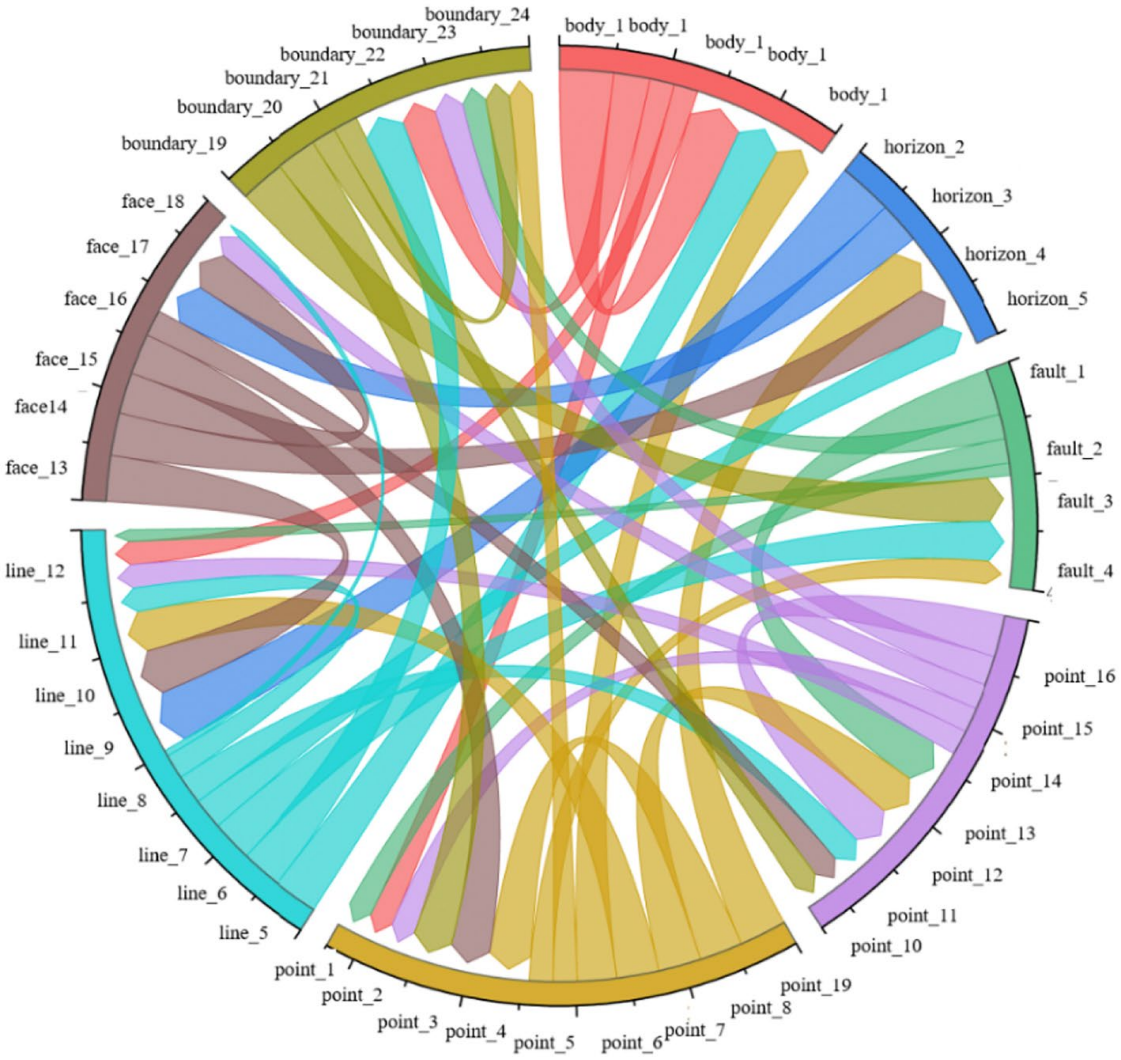
Schematic representation of a knowledge graph in the field of geological structures, illustrating the connectivity among various nodes. Different colors are used to represent the distinct types of edge connections between nodes.

The key pattern mining research of the geological structure knowledge graph is completed using the existing approximate subgraph matching method applied to the actual geological structure knowledge graph. The data set of the geological structure knowledge graph includes intersection point entities, intersection line entities, subsurface entities, and geological block entities. The entities' attributes comprise geological horizon and fault attributes. The relationship types between entities include topological location relations such as inclusion, equality, and cover. The key pattern that constitutes the query graph of approximate subgraph matching is formed by these entities. The aim of this study is to query whether there are key patterns in the existing geological structure knowledge graph through the study of approximate subgraph matching. Expert interaction is then completed to construct a complete geological structure knowledge graph. Figure 2 illustrates the process of key pattern mining of the knowledge graph, and the existing key pattern of geological structure is shown in Fig. 3. The study focuses on key pattern mining through approximate subgraph matching. The knowledge graph is derived by reasoning through the intersection relationship between the horizon plane, fault plane, and boundary plane. These planes serve as the original data for our data graph, as depicted in Fig. 4.

## Experiment

To answer the following questions, we compare our method with state-of-the-art learning methods and exact methods on the task of approximate subgraph matching: Q1: How effective and efficient is our method compared to state-of-the-art learning methods and exact methods in terms of accuracy and speed? Q2: How well does our method perform in approximate subgraph matching, considering node, edge, and structural properties? Q3: To what extent does the proposed our method adapt to noise and unbalanced graph sizes in both the query and target graphs? Q4: How effective is the proposed our method for mining key patterns in knowledge graphs?

### Dataset

To assess our method's ability to identify graph-pair matching relationships in the knowledge graph, we utilised four open graph datasets: Tumblr_ct1^34^, DBLP^35^, Facebook^34^, and Twitter^36^. The specific details are presented in Table 1. Each original graph in the dataset and a randomly selected connected subgraph from the former were treated as a sample pair for each dataset. We then used VF2^6^ to calculate the true matching matrix. Four publicly available datasets and our domain dataset (geological structure modeling) were used to evaluate the effectiveness of the new approximate subgraph matching model. The model's performance in the real world was measured using our domain dataset, which consisted of 773 nodes, 4278 edges, 6 node labels, 8 edge labels, and 7 enumerated key patterns.

**Table 1:** Dataset. avg| G |and avg | Q |are the average size of the data graph and the query graph.

	Tumblr_ct1		DBLP	Facebook	Twiter
Graphs		373	19,456	995	144,033
Avg.node		53.11	10.48	95.72	4.03
Avg.edge		71.63	19.65	101.7	4.98

Field data collection, specifically geological structure modeling, is used to measure the real-world effects of a model. The original dataset is presented as a figure for each sample. For the purposes of this study, each graph pair must contain a data graph and a query graph. To create a graph pair from a single graph in the dataset, we randomly select a graph from the original dataset as the data graph G. Then, we randomly select a connected subgraph from G as the query graph Q. Finally, we use the VF2 algorithm to calculate the truth matching matrix. We repeat this process several times to form the processed dataset. Therefore, the model does not have access to the graph pairs in the test set during the training phase.

### Evaluation index

The performance of node classification is evaluated based on accuracy, F1-score, and running time. Additionally, the accuracy of node-to-node matching and efficiency are also considered.

Accuracy: The ratio of the number of correctly matched nodes in each graph to the total number of nodes in the graph

14 $$accuracy = \frac{NOCC}{{TNON}}$$

where NOCC and TNON represent the number of correctly matched nodes and the total number of nodes in the graph respectively.

F1-score:

15 $$F_{1} = \frac{2*P*R}{{P + R}}$$

P represents precision, which is the ratio of correctly discovered node matches to all discovered node matches. R represents recall rate, which is the ratio of correctly discovered node matches to all correct node matches.

Running Time: We also use the running time to evaluate the efficiency of models.

### Efficiency and accuracy analysis

**Figure 5:**
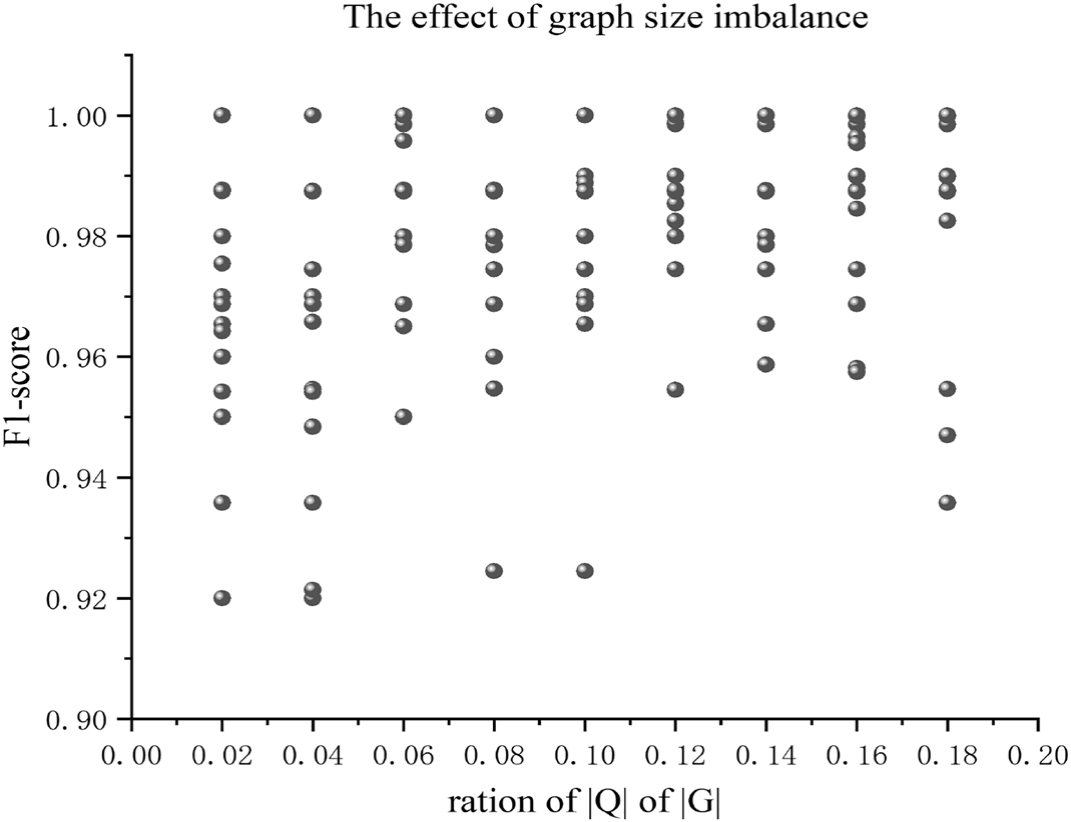
Ratio of query graph to target graph, abscissa shows the F1-Score of approximate subgraph matching in the state of extreme imbalance between query graph and target graph. It can be seen from the figure that the accuracy value is generally higher when the difference between the query graph and the target graph is small than when the difference between the query graph and the target graph is large.

Table 2 shows that our model's prediction speed is relatively fast, even for complex data. We can quickly achieve approximate subgraph matching. When compared to the accurate method and using statistical methods for contrast, we found that our algorithm is faster than the VF2 algorithm and VELSET in terms of time efficiency. It is also comparable to VF3^37^. Figure 7 shows that a low F1-Score results when there is a significant difference between the size of nodes and edges. In Fig. 5, our method effectively handles the imbalance between the size of the node label query graph and target graph. Table 2 shows that our proposed method achieves accuracy comparable to that of VF3 approximate subgraph matching. In addition, our method significantly improves computational efficiency compared to both the statistics-based method and VF2 algorithm. These results indicate that our method is effective in improving accuracy, particularly for large knowledge graph networks.

**Table 2:** Average running efficiency when the number of query graph nodes is 25,and it is compared with the accurate methods (VF2 and VF3).

Model	Tumblr_ct1	Protein	DBLP
VF2	0.76	54	87
VF3	0.006	0.03	7.76
VELSET	17	14	36
Ours	0.0032	0.05	0.76

We compared our method with state-of-the-art (SOTA) learning methods and exact methods in terms of accuracy and efficiency. Accuracy measures the model's ability to match subgraphs accurately, while efficiency measures the speed of the matching process. Our experimental results demonstrated that our method achieved competitive accuracy compared to SOTA-based learning methods while exhibiting improved efficiency. The graph attention mechanism used in our method effectively captures node and edge features, enabling accurate and efficient subgraph matching.

To assess our method's adaptability to noise and unbalanced graph sizes, we conducted experiments using noisy and unbalanced query and target graphs. The results (Fig. 5) showed that our method exhibited robustness in the presence of noise, maintaining its subgraph matching accuracy. Additionally, our method demonstrated the ability to handle unbalanced graph sizes by effectively aligning subgraphs despite differences in size. This adaptability highlights our method's capability to handle real-world scenarios where noise and graph size imbalances are common.

### Comparative experiments

Table 3 compares our existing method with four benchmarks: RDGCN^32^, NeuralMatch^33^, and other deep learning methods. The proposed method achieves a slightly higher F1-Score accuracy than traditional deep learning-based methods. Relational graph neural networks can achieve approximate subgraph matching and better integrate the features of nodes and edges. The graph attention mechanism enables the method to effectively capture the internal structural patterns of the knowledge graph. The proposed method enhances the utilization of nodes, edges, and structural attributes in the matching process, leading to improved subgraph matching performance.

**Table 3:** The F1-scare of three baseline and the proposed model on three datasets.

Model	Facebook	DBLP	Twitter	Protein
RDGCN	0.843	0.745	0.764	0.845
NeuralMatch	0.855	0.877	0.786	0.965
Ours (node)	0.955	0.934	0.928	0.956
Ours (edge)	0.943	0.956	0.954	0.954
Ours (structure)	0.954	0.946	0.923	0.934
Ours (all)	0.964	0.973	0.966	0.962

**Figure 6:**
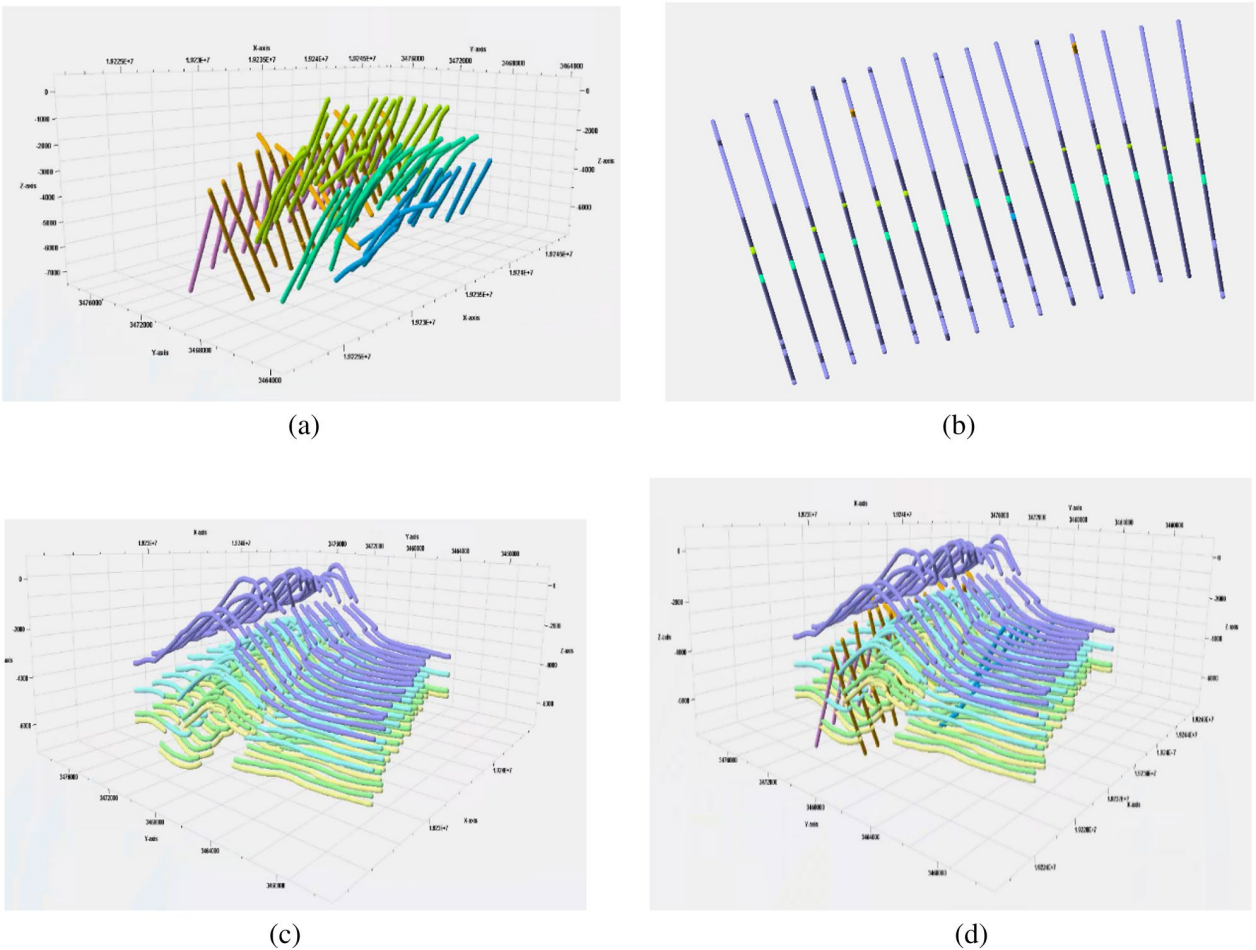
The following data visualizations are presented: (**a**) fault data of a working area, (**b**) 2D visualization of a working area, and (**c**) horizon data of a working area. Additionally, interpretation data of a working area is provided, including the horizon plane and fault plane.

**Figure 7:**
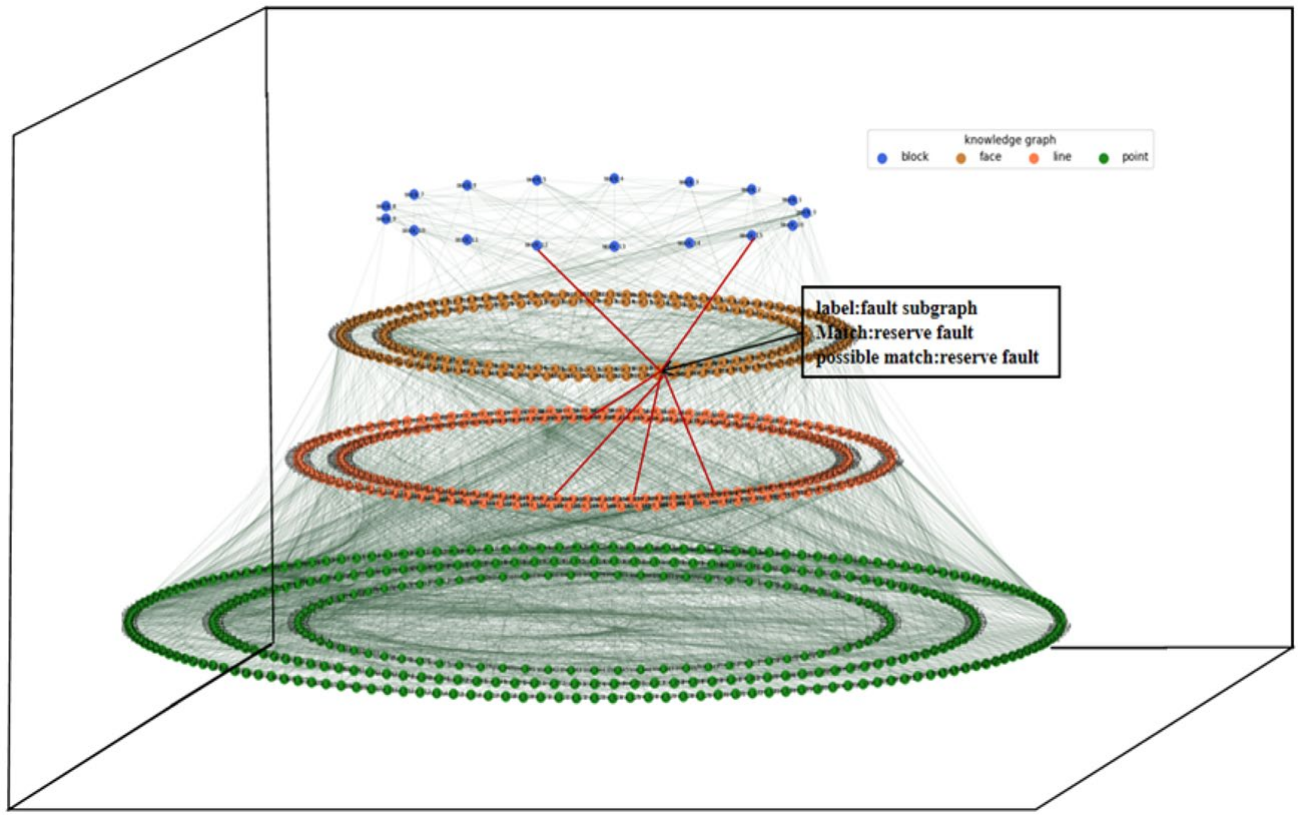
Visualization of key pattern mining of knowledge graph.

**Figure 8:**
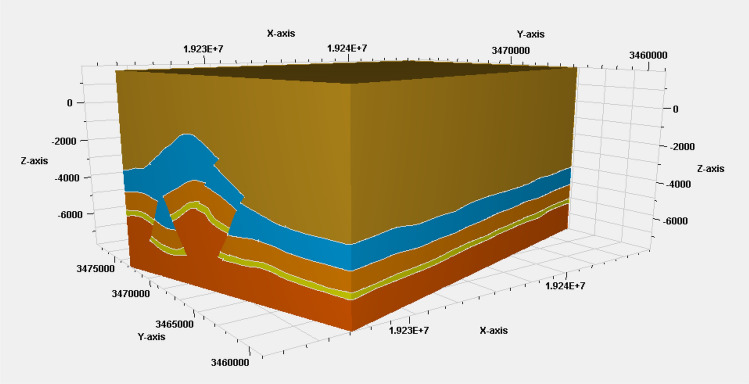
3D modeling of an actual work area.

Figures 7 and 8 illustrate the visual representation of the existing pattern mining by applying the model to the actual seismic data (Fig. 6) knowledge graph research. The graph's layered model represents the cross-layer structure of the knowledge map. Key pattern mining of the knowledge graph is achieved through mouse interaction with the domain data set. This involves matching approximate subgraphs and mining possible structural patterns on the existing knowledge graph.

The subsequent 3D geological modeling is then guided, improving the efficiency of expert interaction. This proves that the existing method can be used for key pattern mining research. However, it is essential to validate the knowledge graph by matching it against expert cognition. This is where approximate subgraph matching techniques come into play. By mining key patterns from the knowledge graph, it becomes feasible to identify potential discrepancies or inconsistencies between the graph and expert knowledge. Through a process of comparison and analysis, the knowledge graph can be modified and refined to better align with the expectations and expertise of domain specialists. The application of approximate subgraph matching in the mining of key patterns not only aids in identifying inconsistencies but also assists in discovering valuable geological structural knowledge that may have been previously overlooked. By revealing these key patterns, it is possible to enhance the knowledge graph with more precise and relevant information, thereby improving its usability and effectiveness in supporting geological modelling and exploration activities.

With the help of a knowledge graph, we can create a three-dimensional model of complex geological structures. By adding constraints from the knowledge graph to the modeling process, we can estimate the intersection lines of the three-dimensional model more accurately (Fig. 7). Once we've determined these intersection lines, we can then reconstruct the entire three-dimensional geological structure using surface reconstruction methods. Figure 8 shows an example of a three-dimensional geological model that can be guided by a knowledge graph^38^.

## Discussion

The method for approximate subgraph matching based on graph neural networks is used to extract key patterns in the geological structure knowledge graph. However, due to the limited number of labelled data and high uncertainty of existing domain datasets, the accuracy of the obtained results cannot be fully guaranteed under a given uncertain dataset. To obtain more accurate data, it is necessary to clean the original data. Improving the accuracy of approximate subgraph matching can be achieved by considering node, edge similarity and graph structure. However, this can also increase the difficulty of key pattern mining. Geological structures are known to have complex topological and multi-scale features, which require handling a large number of variations and differences during subgraph matching. Defining and measuring the similarity of subgraph structures is a challenging task that may require the involvement of domain experts and the integration of domain knowledge.

Simultaneously considering the similarity of nodes, edges, and subgraph structures increases the difficulty of key pattern mining in knowledge graphs but improves the accuracy of approximate subgraph matching. However, this approach may not be as efficient as other learning-based methods. However, the efficiency may decrease when the query graph and the target graph differ significantly.

## Conclusions

This paper proposes an approximate sub-graph matching method to study the key patterns of geological structure knowledge graphs. Traditional approximate sub-graph matching mainly considers node and structural features, without taking into account edge labels. To improve the accuracy of approximate sub-graph matching, we introduce the matching of edge labels and use an adaptive edge deletion mechanism to ensure structural similarity. In addition to verifying the results of approximate sub-graph matching in the existing data set, we have included a real data set for verification. This approach enables the research of approximate sub-graph matching on the domain knowledge graph, the mining of key patterns in the geological structure knowledge graph, and the improvement of knowledge interaction efficiency.

## Data Availability

The datasets generated and/or analysed during the current study are not publicly available due [REASON WHY DATA ARE NOT PUBLIC] but are available from the corresponding author on reasonable request.
